# Factors related to disagreement between self-reported versus objective measurement of allergen sensitization at a tertiary pediatric center in Beijing, China

**DOI:** 10.1186/s12887-020-02148-z

**Published:** 2020-05-28

**Authors:** Qing Miao, Li Xiang, Hui Guan, Yongge Liu, Zhen Li, Yixin Ren, Wei Xu

**Affiliations:** Department of Allergy, Beijng Children’s Hospital, Capital Medical Univeristy, National Center for Children’s Health, Beijing, 100045 People’s Republic of China

**Keywords:** Sensitization, Aeroallergen, Pediatric, Animal dander, Pollen, Dust mite, Cockroach

## Abstract

**Background:**

The objectives of present study were to examine the level of disagreement between self-reported and objective measurement of sensitization to common inhalant allergens, and to explore the potential risk factors that might contribute to this discrepancy.

**Methods:**

A total of 215 children were enrolled from pediatric clinics at a tertiary pediatric center in Beijing, China. A survey questionnaires regarding self-perceived sensitization was completed by participants’ parents/caregiver, meanwhile, skin prick testing(SPT) was performed as objective assessment of sensitization. Extent of agreement between self-reported versus SPT-measured sensitization to individual allergen was calculated using Cohen’s kappa (κ) coefficient. Multivariable regression analyses were used to determine the factors associated with discrepancy between self-reported and objective measurement of sensitization.

**Results:**

119(55.3%) patients have reported to be sensitized to at least one of inhalant allergen, whereas 167(77.7%) patients had a positive skin testing response. Agreement between self-perceived and actual aeroallergen sensitization was moderate for mites(κ = 0.518) and grass pollen mix(κ = 0.451), moreover, fair agreement was observed for mold(κ = 0.316) and cockroach(κ = 0.297), respectively. There was a least agreement between perceived and actual sensitization observed for pet dander, with a kappa coefficient of 0.005. Subjects’ age, atopy history, ownership of pet may increase the risk of disagreement, moreover, background factors of informant, like: age, education level, and the relationship with enrolled subjects, were linked to the incidence of disagreement between self-reported sensitization in comparison with SPT results.

**Conclusion:**

Questionnaire-based self-assessment is easy way to collect clinical information on allergen sensitization; however, the accuracy of questionnaire-derived information is more likely to be influenced by respondent’s background factors. The information from the questionnaire report is considered to be more reliable when in combination with objective assessment of sensitization, including blood IgE testing and SPT.

## Background

Diagnosis and management of allergic disorder often begins with identifying allergen trigger, however, an inadequate recognition of allergen exposure can lead to incorrect avoidance and inadequate disease control [1]. Allergen laboratory testing, such as skin prick testing (SPT) and serum-specific IgE (sIgE) measurement, are strongly recommended in pediatric practice as an objective assessment of sensitization [2, 3]. However, under some special circumstances, a self-administrated survey questionnaires is applied to get a quick grasp on patients’ clinical history during a short period of time due to its time-friendly and cost-efficient advantage [4]. Accordingly, the validate of questionnaire report of allergy-related outcomes should be carefully examined to ensure the accuracy of information. Although a number of studies had previously reported the accuracy of questionnaire-derived data largely depend on the respondents’ knowledge level and their willingness in replying [5], little is known concerning how well questionnaire-derived allergy history actually agrees with objective evaluation index. For this purpose, the level of agreement between questionnaire-based and SPT-measured sensitization to common 5 inhalant allergens was examined at a tertiary pediatric center in Beijing, and the potential risk factors related to this disagreement was explored as well.

## Methods

### Study design and population

The study has a cross-sectional design. Survey participants were selected from patients presenting classical respiratory symptoms (like: sneezing, watery nasal discharge, cough, nasal obstruction) and seeking for medical advice for the first time between May 2015 and November 2017 in pediatric allergy outpatient clinic, Beijing Children’s Hospital. The diagnosis and assessment of asthma was evaluated according to the Global Initiative for Asthma (GINA) criteria, and allergic rhinitis was diagnosed according to the ARIA [6, 7]. Exclusion criteria were as following: (1)Having received standard treatment protocol for allergic diseases(asthma, allergic rhinitis); (2)refuse to complete survey questionnaire; (3)information missing or logic error in a questionnaire; (4)withdrawal from the study; (5)having receiving skin testing previously; (6)children who had taken anti-histamine medication within 7 days prior to SPT; (7)refusal or intolerance to complete skin testing.

This study was approved by Ethical Review Board of Beijing Children’s Hospital, and written informed consent was obtained from all participants, who were older than 6 years of age, or their parents.

### Questionnaire and definition of self-reported sensitization

A self-administered survey questionnaire was required to be finished by parents/caregivers prior to skin prick testing, the full details of allergen exposure survey questionnaire were shown in Additional file 1. The questionnaire was composed of 2 parts. Part I general demographic information of study subjects, including child’s age, place of birth, race/ethnicity (Han/Other), clinical history, family history, living environment, feeding mode (Breast feeding/Mixed feeding), early exposure to antibiotic drugs, delivery mode(Natural/Cesarean section), full-term birth(Yes/No). Respondents’ gender, age, education level, and relationship between respondents and subjects were also recorded. Part II was designed to identify self-reported sensitization by asking following questions: (1)“Do you think that your children are allergic to any of the following allergens?” (2) “Do you think being exposure to any of following allergens could trigger or cause allergic symptoms that bother your children?” (3)“What do you expect the allergy skin tests to be positive for?” The tested allergen included: house dust mite/house dust, pet dander (cat, dog), grass pollen mix, cockroach, and molds/mildew. The answer “Yes” response to above individual allergen given by respondents was then considered as self-reported sensitization for that allergen.

### Objective measurement of allergen sensitization

Sensitization to 6 common inhalant allergens were determined by SPT, including: house dust mites, molds, grass pollen mix (*Artemisia sieversiana*, *Humulusscandens*, *Ambrosia artemisifolia*, *Chenopodium album*), pet dander (cat-dog dander mixture), and cockroach. The procedure of SPT was performed as previously described [8], meanwhile, a positive control (histamine, 10 mg/mL) and negative control (saline) were also included in each test, respectively. SPT was not completed if the child had a severe systemic reaction to an allergen previously or if the child had taken antihistamine on the day of testing. Positive sensitization to allergens was defined as a mean wheal diameter equal or greater to that in positive control.

### Statistical analysis

Statistical analysis was performed using SPSS19.0 software (SPSS, Chicago, IL, USA) and graphs were generated using the prism software (GraphPad, LaJolla, CA, USA). The following tests were used for statistical analysis of the data: student t-tests and 1-way analyses of variance (ANOVAs; with Bonferroni corrections) were used for the comparison of the means of variables with normal distributions, and Mann-Whitney U-tests and Kruskal-Wallis nonparametric ANOVAs were used for variables that were not normally distributed. Level of agreement between questionnaire-based and SPT-measured allergen sensitization was quantified using Cohen’s Kappa coefficient, which are often interpreted as: > 0.80 (very good agreement), 0.61–0.80 (good), 0.41–0.60 (moderate), 0.21–0.40 (fair), and < 0.2 (poor). Potential factors in relation with discrepancy between self-reported and SPT-measured sensitization was determined by a stepwise logistic regression analysis.

## Results

### Baseline characteristics of study population

**Fig. 1:**
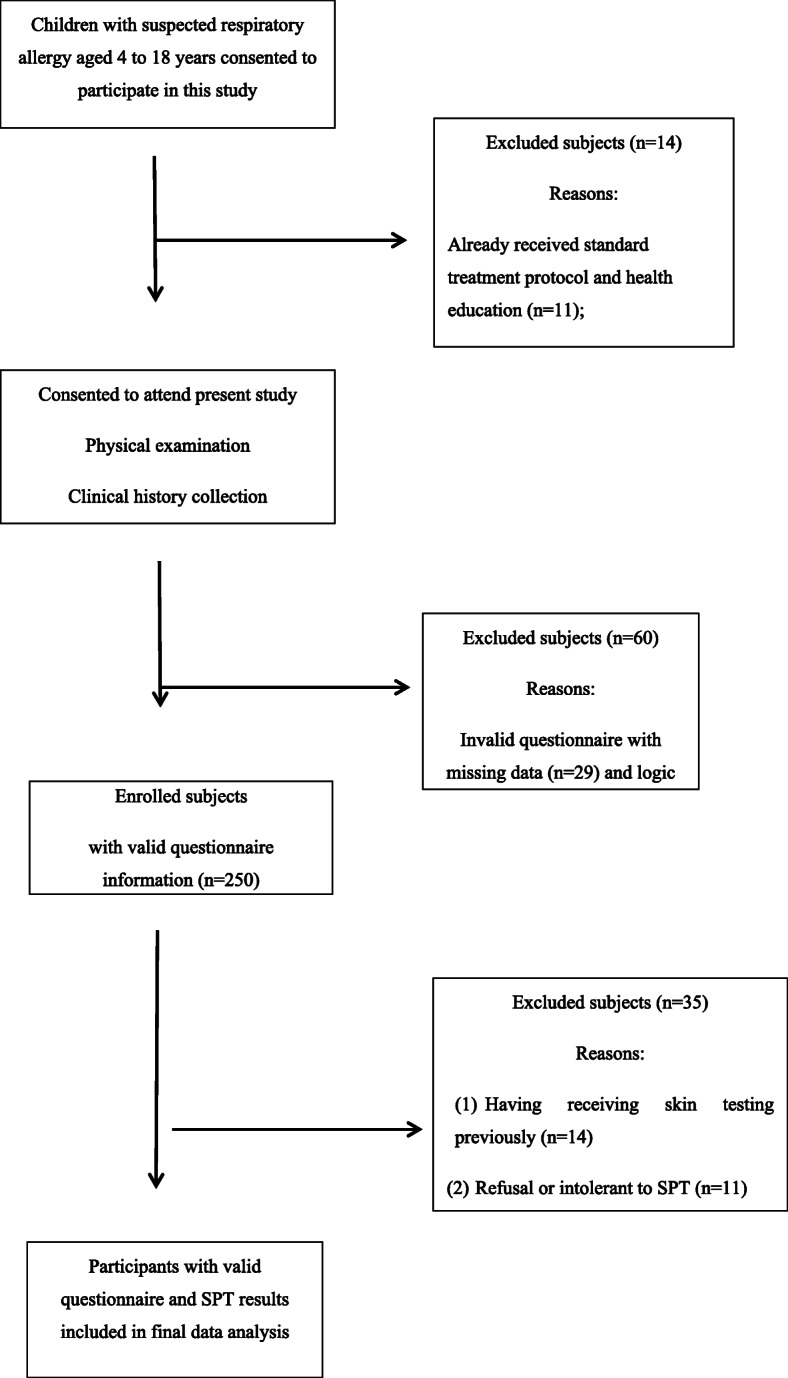
Flow chart of study design

A total of 215 individuals with completed survey questionnaire information and valid SPT results were enrolled into final analysis, and flowchart of the study design was shown in Fig. 1. The median age of subject children was 7.8 years (range 4 to 12 years). The baseline characteristics of study participants are presented in Table 1, the majority of participants were boys(51.2%) and belongs to the Han ethnic group(68.8%). Of 215 subjects who participated, 48.8%(*n* = 105) patients were diagnosed only with asthma, 21.4% (*n* = 46) patients had allergic rhinitis, and 29.8% (*n* = 64) patients who were diagnosed with both asthma and allergic rhinitis.

**Table 1:** Baseline characteristics of participating subjects

Characteristics	Numbers	%
**Sex:** Boys	110	51.2%
**Race/ethnicity:** Han	148	68.8%
**Place of birth:** Urban	134	62.3%
**Doctor-diagnosed allergic disorders**		
Asthma	105	48.8%
Allergic rhinitis	46	21.4%
Asthma and allergic rhinitis	64	29.8%

### Comparison between self-reported and SPT-measured sensitization

**Fig. 2:**
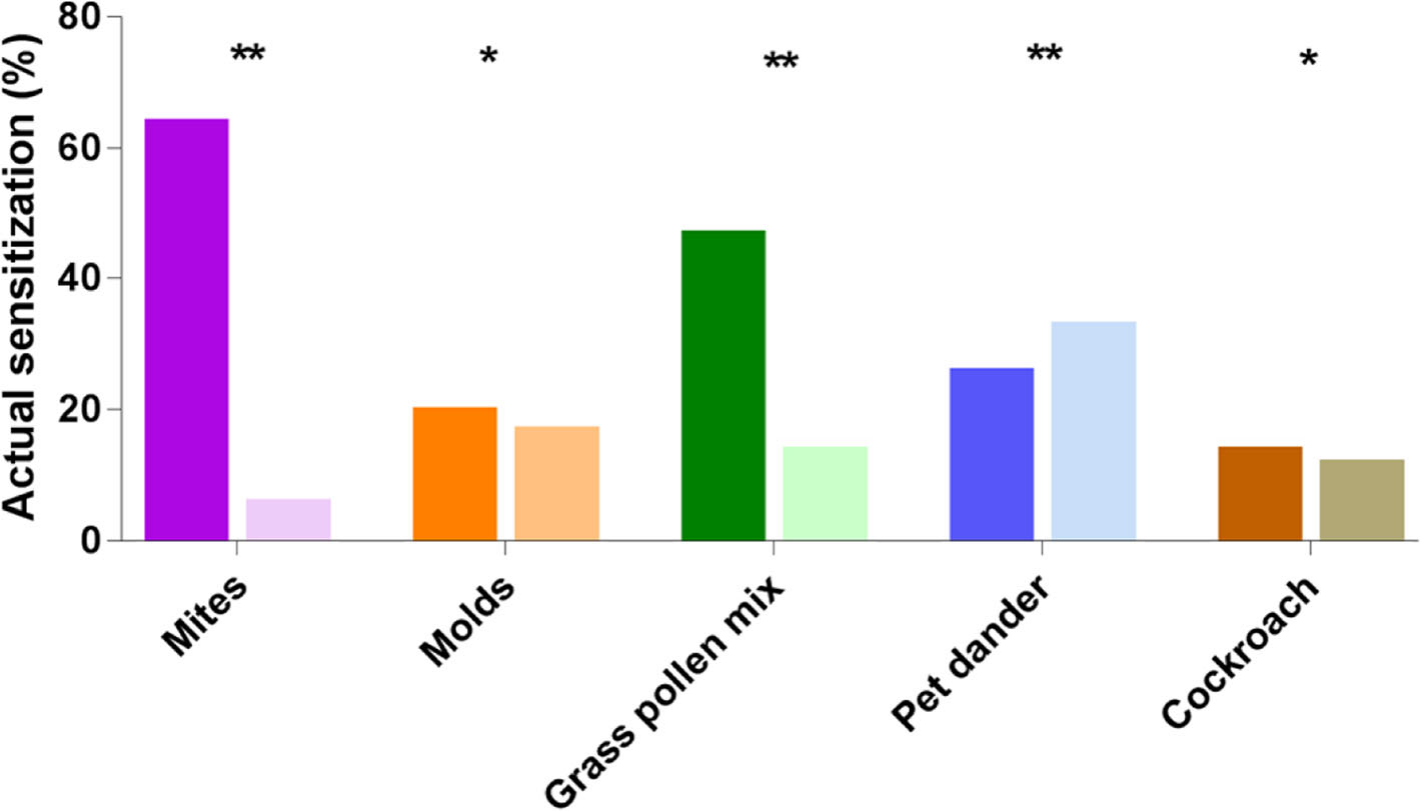
SPT-measured sensitization rates to each allergen between participants with or without self-reported sensitization. The difference in SPT-measured sensitization rates to individual allergen was compared between participants with self-reported sensitization (darker bar) and without self-reported sensitization(lighter bar). *P < 0.05, **P < 0.001

119(55.3%) patients have reported to be sensitized to at least one of inhalant allergen, whereas 167(77.7%) patients had a positive skin testing response. The inter-group difference between self-reported and SPT-confirmed sensitization is significant(*P* < 0.05). Of children with self-perceived sensitization, the actual prevalence in study subjects confirmed by SPT was highest to mites(71.7%), followed by grass pollen mix(63.7%), pet dander(43.7%), molds(39.5%) and cockroach(27.5%). Figure 2 presented the comparison of SPT-measured sensitization rates to each aeroallergen between subjects with and without self-perceived sensitization, interestingly, 62.5% subjects with no self-reported sensitization were still sensitized to at least 1 aeroallergen. Although the prevalence of actual sensitization among children without self-perceived sensitization was significantly lower than their counterparts who perceived allergen sensitization, the SPT-measured sensitization rates within those nonperceivers was highest to pet dander(34.3%), molds(18.1%), grass pollen mix (14.9%), cockroach(12.6%) and mites(7.0%).

### Extent of agreement between self-reported and SPT-measured sensitization

The self-reported and actual SPT-measured sensitization rates to individual aeroallergen were listed in Table 2. House dust mite(76.3%), grass pollen mix(66.5%), and pet dander(61.3%) were the most commonly reported allergens by questionnaire respondents, however, which were significantly less than SPT-confirmed sensitization ratios(mites:71.7%, grass pollen mix:63.7%, pet dander:43.7%). It is surprising that a significantly higher SPT-proven sensitization rates to mold allergen(39.5%) than that verified by self-reported data(30.7%), and the difference was significant (*P* < 0.05).

**Table 2:** Agreement between self-reported versus SPT-measured allergen sensitization

Sensitized to	SPT-measured sensitization (+)		SPT-measured sensitization (+)		SPT-measured sensitization (−)		SPT-measured sensitization (−)		Kappa (95% CI)		Strength of agreement^a^
	Self-reported sensitization (+)		Self-reported sensitization (−)		Self-reported sensitization (+)		Self-reported sensitization (−)				
	n	%	n	%	n	%	n	%			
**≥1 allergen**	81	37.7	34	15.8	45	20.9	55	25.6	0.256	(0.122–0.390)	Fair
**Mites**	139	64.7	15	7.0	25	11.6	36	16.7	0.518	(0.381–0.643)	Moderate
**Grass pollen mix**	105	48.8	32	14.9	38	17.7	40	18.6	0.451	(0.321–0.570)	Moderate
**Molds**	46	21.4	39	18.1	30	9.3	100	46.5	0.316	(0.188–0.445)	Fair
**Cockroach**	32	14.9	27	12.6	36	16.7	120	55.8	0.297	(0.155–0.437)	Fair
**Pet dander**	58	27.0	74	34.3	36	16.7	47	21.9	0.005	(−0.115–0.133)	Poor

^a^ Level of agreement between questionnaire-based and SPT-measured allergen sensitization was quantified using Cohen’s Kappa coefficient, which are often interpreted as: > 0.80 (very good agreement), 0.61–0.80 (good), 0.41–0.60 (moderate), 0.21–0.40 (fair), and < 0.2 (poor)

Next, the level of agreement between self-reported versus SPT-measured sensitization rates was examined using kappa coefficient. As shown in Table 2, overall, there was fair agreement between perceived and actual sensitization to at least 1 aeroallergen (κ = 0.256). Further analysis revealed moderate agreement with respect to mites(κ = 0.518) and grass pollen mix(κ = 0.451), and fair agreement observed for pet dander(κ = 0.316) and cockroach(κ = 0.314). Finally, a least agreement between perceived and actual sensitization was observed for pet dander, with a kappa coefficient of 0.005.

### Background factors in association with disagreement between self-reported versus SPT-measured allergen sensitization

To determine which subjects’ factors were related to the disagreement between self-reported versus SPT-measured sensitization, the background characteristics of participants with or without discrepancy were summarized in Table 3. There were no differences among these 2 groups with respect to child’s gender, race/ethnicity, location, BMI index, antibiotic usage, feeding mode, birth mode, and full-term birth. However, a significant difference was observed with respect to child’s age, showing a higher incidence of disagreement occurred in subjects with higher age than those participants under 7 years(*P* < 0.001;OR = 2.59; 95%CI, 1.63 to 3.37). In addition, an atopy family background increased probability of disagreement in comparison with those without it (OR = 1.18, *P* < 0.001; 95%CI, 1.11 to 2.27). besides, whether keeping pets at home was related to discrepancy, showing that children with a pet at their bedroom were 1.15 times more likely to cause discrepancy upon sensitization when comparing with those without pets inside their home(*P* < 0.001; 95%CI, 1.06 to 1.25). Meanwhile, as shown in Table 4, the potential impacts of respondents’ background factors that might contribute to the disagreement were taken into consideration, including informants’ age, gender, education level, and the relationship with enrolled subjects. No significant difference was observed in respondents’ gender regarding the incidence of discrepancy. However, elderly respondents (≥50 years) were 2.89 times more likely to have disagreement than those under 40 years(*P* < 0.001; 95%CI, 1.34 to 3.73). Moreover, our study revealed respondent with equal or less than medium education level were 2.08 times more likely to present disagreement concerning allergen sensitization evaluation compared with those with a higher educational status (*P* < 0.001; 95%CI, 0.41 to 3.16). In present study, 71.6% of the questionnaires were completed by child’s parents and 28.4% by caregiver. Our results showed that 1.16 times more likely to cause disagreement when the questionnaire was completed by children’s other caregivers(*P* < 0.001; 95%CI, 1.04 to 1.33).

**Table 3:** Background characteristics of participants and respondents related to discrepancy

Factors		Total	With discrepancy		Without discrepancy		Univariate		Multivariate	
			n	%	n	%	OR (95%CI)	P	OR (95%CI)	P
**Gender of child**	Boys	135	31	49.2	79	52.0	0.90 (0.50–1.61)	0.712	–	–
	Girls	80	32	50.8	73	48.0				
**Age of child (yrs)**	0-3 yrs	33	10	14.1	23	16.0	–	–	**Ref**	**–**
	4-6 yrs	78	33	46.5	45	31.3	1.90 (0.95–2.53)	< 0.001	**1.75 (0.84–2.63)**	**< 0.001**
	7-17 yrs	104	38	53.5	66	45.8	2.33 (2.05–3.49)	< 0.001	**2.59 (1.63–3.37)**	**< 0.001**
**Ethnicity of child**	Han	148	45	67.2	103	69.6	0.89 (0.48–1.06)	0.722	–	–
	other	67	22	32.8	45	30.4				
**BMI index**	Normal weight	189	114	88.4	75	87.2	1.12 (0.49–2.56)	0.80	–	–
	Overweight and Obesity	26	15	11.6	11	12.8				
**Location**	Rural	81	11	33.3	70	38.5	0.80 (0.37–1.75)	0.58	–	–
	Urban	134	22	66.7	112	61.5				
**Family history of atopy**	No	76	12	15.2	64	47.1	1.21 (1.14–2.41)	< 0.001	**Ref**	**< 0.001**
	Yes	139	67	84.8	72	52.9			**1.18 (1.11–2.27)**	
**Pet keeping**	No	132	34	34.3	98	84.5	1.18 (1.04–1.36)	< 0.001	**Ref**	**< 0.001**
	Yes	83	65	65.7	18	15.5			**1.15 (1.06–1.25)**	
**Antibiotic use (during first 3 months)**	No	158	64	74.4	94	72.9	1.01 (0.58–2.01)	0.80	–	–
	Yes	57	22	25.6	35	27.1				
**Feeding mode**	Breast feeding	137	43	63.2	94	63.9	0.97 (0.53–1.76)	0.92	–	–
	Mixed feeding	78	25	36.8	53	36.1				
**Birth mode**	Natural	132	28	59.6	104	61.9	0.89 (0.47–1.79)	0.72	–	–
	Cesarean section	83	19	40.4	64	38.1				
**Full-term birth**	Yes	183	75	83.3	108	86.4	0.79 (0.37–1.67)	0.53	–	–
	No	32	15	16.7	17	13.6

**Table 4:** Background characteristics of participants and respondents related to discrepancy (continued)

Factors		Total	With discrepancy		Without discrepancy		Univariate		Multivariate	
			n	%	n	%	OR (95%CI)	P	OR (95%CI)	P
**Gender of respondents**	Male	45	13	23.2	32	20.1	1.12 (0.58–1.49)	0.63	–	–
	Female	170	43	76.8	127	79.9				
**Age of respondents (yrs)**	20–30	67	24	28.2	43	33.1	–	–	**Ref**	**–**
	30–40	90	22	25.9	68	52.3	2.90 (1.95–3.34)	< 0.001	**2.75 (1.48–3.63)**	**< 0.001**
	40 and above	58	39	45.9	19	14.6	3.33 (2.05–3.95)	< 0.001	**2.89 (1.34–3.73)**	**< 0.001**
**Educational status of respondents**	Medium and high	76	45	44.6	31	27.2	2.15 (1.22–3.80)	0.008	Ref	< 0.001
	Low	139	56	55.4	83	72.8			2.08 (0.41–3.16)	
**Relationship with child**	Parents	154	32	42.7	122	87.1	1.21 (1.06–1.22)	< 0.001	Ref	< 0.001
	Other caregivers	61	43	57.3	18	12.9			1.16 (1.04–1.33)	

Note: Education was categorized as low (received only primary education or no education), medium (finished secondary school or high school) and high (graduated from college or university)

## Discussion

Establishment causal relationship between allergen exposure and clinical symptoms are considered as the crucial step to management of allergic disorders [9, 10]. In our real-world clinic practice, it is recommend that children’s parents/caregivers are invited to finish a survey questionnaire before seeing an allergist. The questionnaire was modified from the ISAC questionnaire and designed to collect detailed information on patients’ clinical symptoms, family history, living environment, lifestyle habits, and past exposure history. By answering the questionnaire, parents can conveniently evaluate or summarize the potential triggers for their children’s symptoms, but also improving the doctor-patients communication efficiency especially during a short time period of clinic visiting. However, it is not uncommon that some disagreements occurred between parents self-reporting and objective measurement of sensitization status, which greatly drew our interests to explore the potential factors that could contribute for this discrepancy.

Based on our results, dust mite has been verified to be the most-commonly sensitized aeroallergen by both respondents’ self-reporting and SPT measurement, which was line with previous study by Li et al. showing that up to 50% of school-age children with previous history or current symptoms of asthma were sensitized, moreover, the main sensitized allergen was mite [11]. Surprisingly, our results revealed that 62.5% subjects with no perceived allergen sensitization still had high prevalence of actual sensitization. Moreover, of those children who denied any perceived sensitization the type of allergen with highest actual sensitization rate were pet dander(34.3%), followed by molds(18.1%), and cockroach(12.6%). A US study of 253 urban children had reported similar finding that even if a child does not perceive sensitization to any allergen, actual allergen sensitization is still possible, particularly for indoor allergens cockroach [12]. In addition, Li JT et al. study had previously examine the ability of allergic patients to correctly predict SPT results, showing that only 10 to 56% of predictions for skin test result were correct [13]. All above finding may give us a hint that parents were very limited in their ability to correctly identify the allergen(s) to which they are allergic.

Our results revealed a poor agreement for pet dander between self-reporting and SPT measurement (κ = 0.005). Similarly, a previous study by Gehring U et al. had reported questionnaire-reported pets ownership was a relatively weak predictor for the presence of pet allergen in house dust, however, when taking pet contact history druing past 2 years into analysis, the sensitivity of questionnaire-reported could be increased by 10 to 42% [14, 15]. The author explain the reason for long existence of cat dander in househod is caused by the characteristics of cat allergen. Cat dander is a material shed from cats, which is naturally released by the sebaceous glands of a cat such as in the skin, saliva, and fur. Among of them, Fel d1, the main allergen commonly found in a cat’s urine and other body secretions, is very sticky, persistent and capable to glue itself to hair, dust particle(forming a persistent aerosol) and all part of the home. As a results, it is difficult to reduce cat allergen levels in people’s homes completely [16]. Previous study showed that cat allergen can remain distributed throughout the home up to 6 months and in the cat’s bedding for up to 4 years even after removing the cat from bedroom [17]. Compared with the family without a cat, a 10-fold higher cat allergen levels was observed in homes in which had been kept within the past year [18]. Similarly, Nafstad et al. showed that the detectable level of cat and dog allergens were still present in house dust of homes where no pets were kept [19]. Therefore, based on above finding it is rational that more questions on past pet contact history in both inside and outside children’s bedroom should be included in the survey questionnaire, which is believed to be necessary to optimize survey questionnaire, especially information about pet allergen exposure by means of questionnaire-based assessment. Our study revealed that the greatest agreement observed for dust mites and grass pollen, which were inconsistent with the previous studies. A study of rural schoolchildren in Canada had reported a fair agreement was found for reported allergy and sensitization by SPT to cat, but only slight agreement for dust and grass [20]. Another study of 247 asthmatic adults in US found no significant correlation between reported allergy to pollen and wheal size to pollen, and mild correlation for reported pet allergy and SPT wheal size to cat and dog [21]. We assumed the possible reason for the discrepancy might be caused by the differences in study design, sample size, study population, and sensitization assessment method.

Multiple regression analysis revealed a decreased likelihood of discrepancy was related to the presence of atopy history, which was line with previous study reporting a higher level of knowledge about childhood asthma in atopy-phone families [22, 23]. A possible explanation for this phenomenon is that daily living with an atopic family members enables greater learning on the part of parents, encouraging parents to seek related knowledge more actively, which naturally guarantee the accuracy of self-administrated questionnaire information [24]. Educational attainment of parents/caregivers was another significant predictor of discrepancy, based on the finding that less educated parents/caregivers had more likely of disagreement between self-perceived and actual sensitization. A similar finding was reported that parents with lower education level were associated with increased likelihood of asthma occurrence, its severity, and hospitalization indexes, the author assumed the possible reason for that might be caused by the under-recognition of asthma by parents from under developed class [25]. It is natural that parents with a lower socioeconomic status may report the presence of asthma only when there present more severe symptoms or especially when requiring hospitalization to relieve symptom [26]. On the contrast, due to more access to health care knowledge resource, parents with higher education background tend to have correct understanding and comprehensive information about the management and treatment of the disease [27]. Finally, the informant’s age and the relationship between informant and participants are proven to be associated with the disagreement between self-reported and objective measurement of sensitization. Within the elderly informant group, due to lacking of related knowledge to identify allergen triggers or being incapable of recalling with patients’ exposure history comprehensively, it is commonplace that those related to allergy-related skin (e.g. itching, rashes, hives, eczema) symptoms were usually unrecognized and misattributed by children’s supervisors to unhygienic practices (e.g. unwashed clothes or bedding; irregular bathing). Therefore, in future clinical practice it is recommended to choose the subjects’ parents as first choice of respondent to complete the questionnaire. Moreover, some images, video or a combination of above, designed for illustrating allergen trigger and their possible source, should be utilized to decreased the incidence of disagreement between self-reported and objective measurement of sensitization. There are some limitations to our study. First, the majority of participants were hospital-based, which might introduce potential selection bias. Second, the study was small involving only 215 patients, the main results of present analyses should be generalized and validated into a larger population size. Last but not the least, the study was based on the assumption that the allergy skin test results accurately defined the hypersensitivities to each aeroallergen. However, in real-world clinical practice, it is not rare that skin test reactivity is not a good predictor for true allergy, i.e., skin tests may result in a false positive diagnosis of allergy. A positive skin response does not necessarily means the clinical hyper-response to this positive allergen. Therefore, in next step we could include more parameter to get a more objective and comprehensive evaluation of participant’s sensitization status, like: SPT, blood testing or even BAT.

## Conclusion

In summary, even though the questionnaire-based self-assessment is easy way to collect clinical information on allergen sensitization, however, the accuracy of questionnaire-derived information is more likely to be influenced by respondent’s background factors. Both questionnaire-derived and objective measurement of allergen sensitization cannot be used interchangeably to assess IgE-related allergic sensitization in pediatric population, the information from the questionnaire report is considered to be more reliable when in combination with objective assessment of sensitization, including blood IgE testing and SPT.

## Supplementary information

**Additional file 1:Table 1.** Allergen exposure survey questionnaire

## Data Availability

The datasets during and/or analyzed during the current study are available from the corresponding author on reasonable request.

## Abbreviations

*SPT*: Skin prick testing
*sIgE*: Serum-specific IgE
BMI index: Body mass index
CI: Confidence interval
