# Supplementary material for: Factors related to disagreement between self-reported versus objective measurement of allergen sensitization at a tertiary pediatric center in Beijing, China
Source: BMC Pediatr. 2020 May 28;20:259. doi: 10.1186/s12887-020-02148-z (PMC7254731; doi:10.1186/s12887-020-02148-z)
Supplement: Supplementary file 1 — Additional file 1:Table 1. Allergen exposure survey questionnaire [file 12887_2020_2148_MOESM1_ESM.docx]

### **[Additional file](https://static-content.springer.com/esm/art:10.1186/s12887-019-1766-2/MediaObjects/12887_2019_1766_MOESM2_ESM.pdf)**

### **[Table1. Allergen exposure survey questionnaire](https://static-content.springer.com/esm/art:10.1186/s12887-019-1766-2/MediaObjects/12887_2019_1766_MOESM2_ESM.pdf)**

*[This questionnaire can be completed in about 15 minutes. Your answers will be entirely anonymous: there is no identification number on the questionnaire or the reply-paid envelope.](https://static-content.springer.com/esm/art:10.1186/s12887-019-1766-2/MediaObjects/12887_2019_1766_MOESM2_ESM.pdf)*

**[Please return the completed questionnaire to us in the envelope provided or to the address below.](https://static-content.springer.com/esm/art:10.1186/s12887-019-1766-2/MediaObjects/12887_2019_1766_MOESM2_ESM.pdf)**

*[Thank you for your time and co-operation.](https://static-content.springer.com/esm/art:10.1186/s12887-019-1766-2/MediaObjects/12887_2019_1766_MOESM2_ESM.pdf)*

*[Further details about the background to the project are supplied on the last page but if you have any questions about the survey please contact:](https://static-content.springer.com/esm/art:10.1186/s12887-019-1766-2/MediaObjects/12887_2019_1766_MOESM2_ESM.pdf)*

[Qing Miao](https://static-content.springer.com/esm/art:10.1186/s12887-019-1766-2/MediaObjects/12887_2019_1766_MOESM2_ESM.pdf)

[Department of Allergy](https://static-content.springer.com/esm/art:10.1186/s12887-019-1766-2/MediaObjects/12887_2019_1766_MOESM2_ESM.pdf)

[Beijing Children's Hosptial](https://static-content.springer.com/esm/art:10.1186/s12887-019-1766-2/MediaObjects/12887_2019_1766_MOESM2_ESM.pdf)

[Telephone 010-59616158](https://static-content.springer.com/esm/art:10.1186/s12887-019-1766-2/MediaObjects/12887_2019_1766_MOESM2_ESM.pdf)

**[-----------------------------------------------------------------------------------------------------](https://static-content.springer.com/esm/art:10.1186/s12887-019-1766-2/MediaObjects/12887_2019_1766_MOESM2_ESM.pdf)**

### **[Allergen exposure survey questionnaire](https://static-content.springer.com/esm/art:10.1186/s12887-019-1766-2/MediaObjects/12887_2019_1766_MOESM2_ESM.pdf)**

**Part I**

1. **General demographic information of participated patients**

- Age: (yrs)
- Gender: □Boy □ Girl
- Ethnicity □Han □Other
- Place of birth: □rural □urban
- Clincal diagnosis: □asthma(AS) □allergic rhinitis(AR) □ a combination of AS and AR

1. **General demographic information of questionnaire respondents**

- Relationship with the enrolled subjects: □Parents □caregiver
- Age(yrs): □20-30 □30-40 □40 and above
- Education level:

□Low (received only primary education or no education)

□Medium (finished secondary school or high school)

□ High (graduated from college or university).

1. **Clinical history**

- BMI index □ Normal weight □Overweight and Obesity
- Family history of atopy: □No □Yes
- Ownership of a pet: □Yes □No
- Antibiotic use (during first 3 months) □No □Yes
- Feeding mode: □Breast feeding □ Mixed feeding
- Birth mode: □Natural □Cesarean section
- Full-term birth: □Yes □No

1. **Home and living conditions**

- Stuffed toys in child’s bedroom? □Yes □No
- Home has indoor feathered or furry pets? □Yes □No
- Do you dust the child’s bedroom regularly? □Yes □No
- Do you vacuuming the floor of the child’s bedroom ? □Yes □No
- Do you washing the child’s sheets and pillowcases ? □Yes □No
- Do you washing the stuffed toys in the child’s bedroom? □Yes □No
- Mold has been seen or a musty odor has been smelled in the subject child’s bedroom? □Yes □No
- Evidence of cockroaches inside the home? □Yes □No
- Smoker among those who take care of the subject child? □Yes □No

**Part II**

1. **Allergen exposure history**

(1)“Do you think that your children are allergic to any of the following allergens?”

□House dust mite/house dust

□pet dander

□grass pollen mix

□cockroach

□molds/mildew

(2)“Do you think being exposure to any of following allergens could trigger or cause allergic symptoms that bother your children?” □House dust mite/house dust

□pet dander

□grass pollen mix

□cockroach

□molds/mildew

1. “What do you expect the allergy skin tests to be positive for?”

□House dust mite/house dust

□pet dander

□grass pollen mix

□cockroach

□molds/mildew
